# Massive bleeding from a Bricker ileal conduit during FOLFIRINOX therapy in noncirrhotic portal hypertension: suspected oxaliplatin-associated sinusoidal injury and presumed irinotecan-associated conduit mucositis: a case report

**DOI:** 10.1186/s12894-026-02204-3

**Published:** 2026-05-29

**Authors:** Rafał Bogdan Drobot, Tymoteusz Ślósarz, Artur Andrzej Antoniewicz

**Affiliations:** 1 Urology Department, Institute of Medical Sciences, Faculty of Medicine, Collegium Medicum, Cardinal Stefan Wyszyński University in Warsaw, Bursztynowa 2, Warsaw, 04-749 Poland; 2 Department of Urology and Urological Oncology, Multidisciplinary Hospital in Warsaw- Międzylesie, Bursztynowa 2, Warsaw, 04-749 Poland; 3 EAU YAU Robotics in Urology Working Group, Arnhem, The Netherlands

**Keywords:** Case reports, FOLFIRINOX, Hematuria, Irinotecan, Mucositis, Oxaliplatin, Portal hypertension, Sinusoidal obstruction syndrome, Urinary diversion, Varicose veins

## Abstract

**Background:**

Bleeding from an ileal conduit after radical cystectomy is uncommon and is usually attributed to tumor recurrence, infection, stones, or local stomal trauma. Portal hypertensive stomal varices are rare. FOLFIRINOX adds further diagnostic complexity because oxaliplatin may cause sinusoidal endothelial injury and noncirrhotic portal hypertension, whereas irinotecan may damage gastrointestinal mucosa. We report a case of massive hemorrhage from a long-functioning Bricker ileal conduit during FOLFIRINOX therapy.

**Case presentation:**

A 72-year-old man with prior radical cystoprostatectomy with Bricker diversion in 2022, robot-assisted left adrenalectomy in 2023, and pancreatic head adenocarcinoma treated with FOLFIRINOX was admitted in February 2026 because of sudden gross hematuria that rapidly filled the urostomy bag with blood and clot. He had received two FOLFIRINOX cycles. With a height of 170 cm and weight of 70 kg, body-surface area was 1.82 m² by the Mosteller formula. Using the standard FOLFIRINOX oxaliplatin dose of 85 mg/m², the reconstructed cumulative oxaliplatin exposure was approximately 170 mg/m², corresponding to about 310 mg in total. Bleeding began 8 days after the most recent irinotecan-containing cycle. Contrast-enhanced computed tomography showed periportal tumor-related changes with a biliary stent, variceal collaterals adjacent to the conduit, and circumferential thickening of the conduit wall, whereas the upper urinary tracts were nondilated and there was no radiologic evidence of recurrent urothelial carcinoma. Looposcopy demonstrated diffusely inflamed and friable conduit mucosa, but biopsy was not performed. Doppler ultrasonography demonstrated portal vein dilatation with preserved hepatopetal flow and no imaging features of cirrhosis. Platelet counts remained within the reference range, and retrospective review of restaging computed tomography showed no interval splenomegaly; the craniocaudal splenic length was approximately 11 cm and unchanged. Urine culture grew extended-spectrum beta-lactamase-producing Escherichia coli and Enterococcus faecalis, but inflammatory markers remained low and there were no clinical features of sepsis. The most cautious interpretation was a mixed mechanism: portal hypertensive stomal varices, possibly related to pancreatic venous distortion and/or early oxaliplatin-associated sinusoidal injury, compounded by presumed irinotecan-associated conduit mucositis. Bleeding ceased with supportive, anti-inflammatory, antimicrobial, transfusion, and nonselective beta-blocker therapy. By May 2026, conduit bleeding had not recurred, although the patient remained chronically unwell with poor ECOG performance status because of the underlying pancreatic cancer.

**Conclusions:**

Massive bleeding from a Bricker conduit during FOLFIRINOX therapy may be multifactorial. Early portal-phase imaging and portal venous assessment are essential when hemorrhage is disproportionate to routine urinary tract findings. In the absence of histology, catheter venography, and portal pressure measurement, causal language should remain cautious. If bleeding recurs, management should move beyond local measures and consider venous mapping, targeted embolization or sclerotherapy, portal decompression or venous stenting in suitable anatomy, and surgical undiversion to bilateral cutaneous ureterostomies when durable conduit preservation appears unlikely.

## Background

Bleeding from an ileal conduit after radical cystectomy is uncommon and usually prompts investigation for recurrent urothelial carcinoma, a secondary conduit neoplasm, infection, calculi, mucosal trauma, or a technical stomal problem [1–3]. That conventional differential is essential, but it can become too narrow when the conduit is constructed from bowel. A bowel-derived urinary diversion is not only part of the urinary tract. It can also function as an ectopic portal-hypertensive outlet.

Ectopic varices account for a small minority of variceal bleeding episodes, but they can be life-threatening and are often diagnosed late because hemorrhage occurs outside the gastroesophageal territory [4, 5]. A 2024 review focused on stomal variceal hemorrhage in ileal conduit diversion summarized only 27 accessible cases, underscoring the rarity of this entity and the frequency with which diagnosis depends on cross-sectional imaging that demonstrates mesenteric collaterals around the conduit [6]. Once ectopic stomal bleeding is suspected, contemporary guidance emphasizes venous inflow anatomy and the expected durability of local versus decompressive therapy rather than stomal appearance alone [4, 7].

Oxaliplatin also deserves specific attention in patients receiving FOLFIRINOX. In the original FOLFIRINOX schedule, oxaliplatin is administered at 85 mg/m² every 2 weeks together with irinotecan, leucovorin, and fluorouracil [8]. Oxaliplatin-based chemotherapy is associated with sinusoidal endothelial injury, sinusoidal obstruction syndrome, nodular regenerative hyperplasia, splenomegaly, thrombocytopenia, and noncirrhotic portal hypertension [9, 10]. These clinical markers are useful, but they are indirect. Their absence, especially after short exposure, reduces diagnostic certainty but does not fully exclude early sinusoidal injury.

At the same time, irinotecan-containing regimens can cause delayed gastrointestinal mucosal injury through epithelial apoptosis, inflammatory amplification, mucin depletion, and microbial beta-glucuronidase-mediated recycling of the active metabolite SN-38 [11–15]. Supportive-care guidance exists for gastrointestinal mucositis in general, yet no conduit-specific framework has been proposed [12, 13]. Pancreatic malignancy can also generate noncirrhotic portal-hypertensive physiology by compressing, invading, or distorting the portal or splenic venous system, thereby creating collateral pathways that bleed at unusual sites [16, 17]. We report a patient in whom these processes appeared to converge, producing abrupt high-volume hemorrhage from a long-functioning Bricker conduit.

## Case presentation

**Fig. 1:**
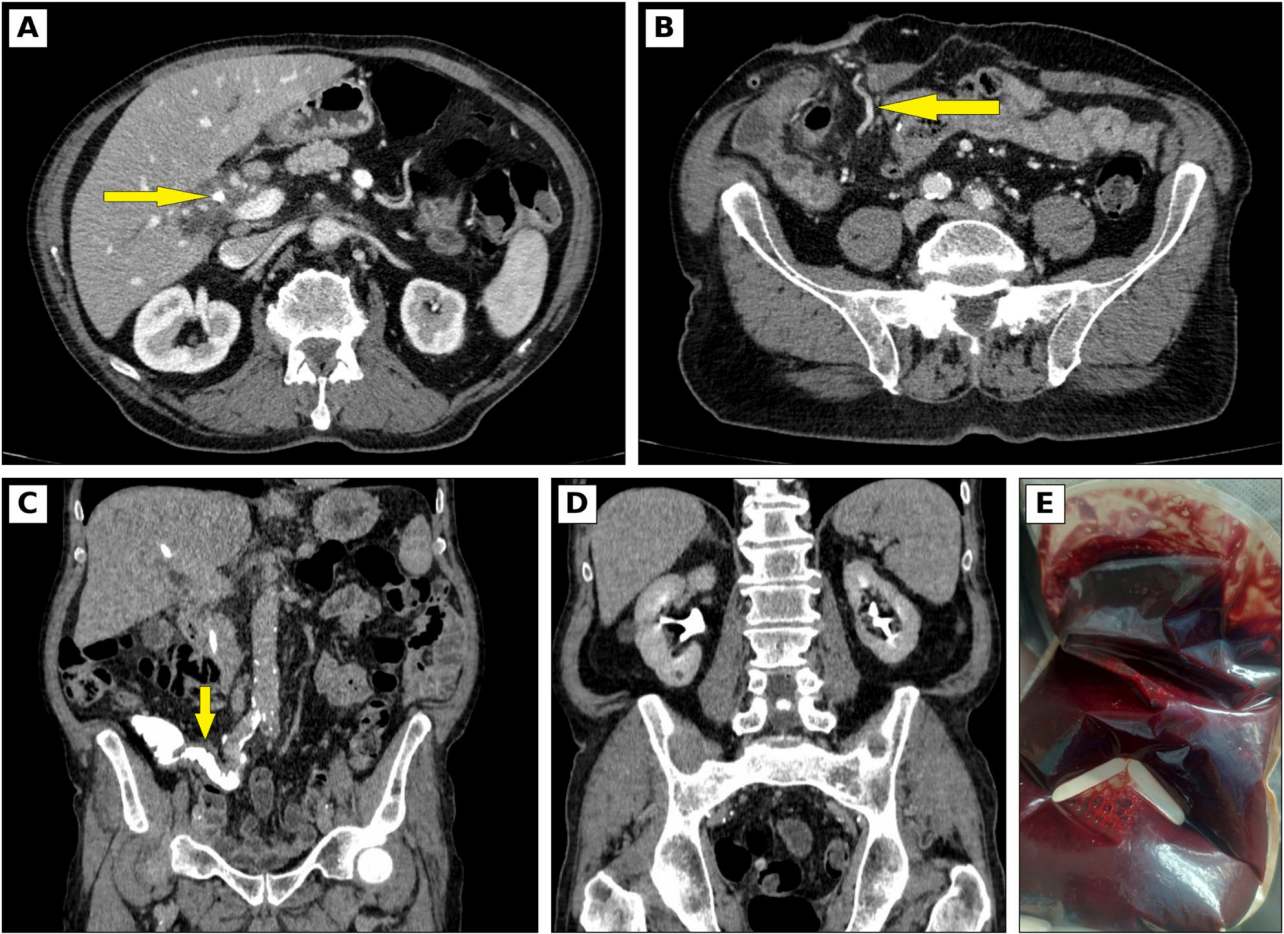
Imaging and clinical appearance of conduit hemorrhage. **A** Axial portal venous-phase computed tomography showing the biliary stent and periportal or pancreatic tumor-related changes. **B** Axial portal venous-phase computed tomography showing venous collaterals adjacent to the Bricker conduit. **C** Coronal urographic-phase reconstruction showing circumferential thickening of the conduit wall. **D** Coronal urographic-phase reconstruction showing nondilated upper urinary tracts without radiologic evidence of recurrent urothelial carcinoma. **E** Urostomy bag filled with gross blood and clot during the index bleeding episode

A 72-year-old man was admitted in February 2026 because of sudden gross hematuria that rapidly filled his urostomy bag with blood and clot (Fig. 1E). Exact admission and discharge dates have been generalized to preserve de-identification. His clinical timeline is summarized in Table 1. He had undergone radical cystoprostatectomy with Bricker ileal conduit diversion for bladder cancer in 2022. In 2023 he underwent robot-assisted left adrenalectomy for a left adrenal mass. In 2025 pancreatic head adenocarcinoma was diagnosed after biliary obstruction, prompting endoscopic retrograde cholangiopancreatography and repeated biliary stent exchanges.

**Table 1:** Timeline of oncologic, portal, chemotherapy, and bleeding events

Date or period	Clinical event	Relevance to current case
2022	Radical cystoprostatectomy with Bricker ileal conduit diversion for bladder cancer.	Created the bowel-derived urinary diversion that later became the bleeding site.
2023	Robot-assisted left adrenalectomy for a left adrenal lesion.	Illustrates complex prior oncologic and surgical history but was not the direct source of hemorrhage.
2025	Pancreatic head adenocarcinoma diagnosed; biliary obstruction treated with ERCP and repeated biliary stent exchanges.	Established the background condition capable of generating noncirrhotic portal hypertension and unusual collateral pathways.
Late 2025 to early 2026	FOLFIRINOX was administered. The patient received two cycles before the bleeding episode. Body-surface area was 1.82 m². Reconstructed standard oxaliplatin exposure was approximately 85 mg/m² per cycle, 170 mg/m² cumulatively, or about 310 mg total.	Provided a plausible oxaliplatin-related portal sinusoidal injury exposure, although the cumulative dose was low and typical indirect markers were absent.
February 2026, day − 8	Most recent FOLFIRINOX cycle, including irinotecan, before admission.	Temporal window compatible with delayed irinotecan-related mucosal toxicity.
February 2026, hospital day 1	Abrupt gross hematuria with clot from the conduit; admission and transfusion requirement.	Index presentation of severe conduit bleeding. Exact dates were generalized for de-identification.
February 2026, hospital day 1	Contrast-enhanced CT showed peri-conduit varices, conduit-wall thickening, and no radiologic upper-tract recurrence.	Supported a mixed venous and conduit-wall mechanism and helped exclude common urologic causes.
February 2026, early admission	Looposcopy showed diffusely inflamed and friable Bricker conduit mucosa without biopsy.	Supported mucosal inflammation but did not prove irinotecan-associated mucositis histologically.
February 2026, early admission	Urine culture grew ESBL-producing Escherichia coli together with Enterococcus faecalis.	Suggested colonization or localized inflammation rather than sepsis as the dominant driver.
February 2026, index admission	Doppler ultrasonography showed portal-vein dilatation with preserved hepatopetal flow and no cirrhosis.	Supported a noncirrhotic portal-hypertensive substrate.
February 2026, discharge	Discharge after conservative stabilization with prednisone taper and propranolol.	Bleeding resolved, but therapy was considered temporizing rather than definitive for varices.
May 2026	No recurrent conduit bleeding had occurred. Overall condition remained chronically poor with poor ECOG performance status due to pancreatic cancer.	Adds short-term follow-up while acknowledging limited prognosis and limited long-term inference.

*Abbreviations*: *CT* Computed tomography, *ECOG* Eastern Cooperative Oncology Group, *ERCP* Endoscopic retrograde cholangiopancreatography, *ESBL* Extended-spectrum beta-lactamase, *FOLFIRINOX* Fluorouracil, leucovorin, irinotecan, and oxaliplatin

Systemic treatment consisted of FOLFIRINOX. The patient had received two cycles before the bleeding episode. He was 170 cm tall and weighed 70 kg, giving a body-surface area of 1.82 m² by the Mosteller formula. Using the standard oxaliplatin dose in FOLFIRINOX, 85 mg/m² per cycle, the reconstructed oxaliplatin exposure was approximately 155 mg per cycle and approximately 310 mg in total, corresponding to 170 mg/m² cumulatively [8]. The most recent irinotecan-containing cycle had been administered 8 days before the bleeding episode. The conduit had functioned without prior hemorrhage since cystectomy. Relevant comorbidities included hyperlipidemia, hepatic steatosis, and active tobacco exposure. Family history, genetic data, and patient ethnicity were not documented in the contemporaneous record.

On presentation he was alert, normothermic, and hemodynamically stable. During internal-medicine consultation his heart rate was 84 beats/min and blood pressure was 97/60 mm Hg. He had no abdominal guarding, no dyspnea, and no clinical cholangitis. Hemoglobin was 6.0 mmol/L (9.7 g/dL) on the first documented evaluation that guided transfusion, and red blood cell support was required. Platelet counts available before and during the admission remained within the local reference interval and did not show progressive or prolonged thrombocytopenia. Key diagnostic findings from the index admission are summarized in Table 2. Urinalysis was uninterpretable because of gross blood. Urine culture later grew extended-spectrum beta-lactamase-producing Escherichia coli and Enterococcus faecalis, but C-reactive protein remained only mildly elevated and procalcitonin was low. In patients with bowel urinary diversion, culture positivity may reflect colonization rather than the primary cause of acute bleeding [11]. There were no clinical features of sepsis and no bowel symptoms suggesting a primary enteric bleeding source.

**Table 2:** Selected diagnostic and chemotherapy-related data during the index admission

Domain	Finding	Interpretation
Body size and dose reconstruction	Height 170 cm; weight 70 kg; body-surface area 1.82 m² by the Mosteller formula. After two standard FOLFIRINOX cycles, reconstructed oxaliplatin exposure was approximately 170 mg/m², or about 310 mg total.	Addresses the requested oxaliplatin exposure. The cumulative dose was low compared with many established oxaliplatin-SOS presentations, so causality remains cautious.
Hemodynamic status	Hemodynamically stable despite profuse stomal bleeding; transfusion was required.	Severity was clinically meaningful, but there was time for diagnostic synthesis before invasive rescue therapy.
Anemia	Hemoglobin 6.0 mmol/L (9.7 g/dL) on the first documented evaluation that guided transfusion.	Consistent with acute blood loss from the conduit.
Platelet trend	Available platelet counts remained within the local reference interval before and during admission, without progressive or prolonged thrombocytopenia.	Argued against fully developed oxaliplatin-related portal hypertension with hypersplenism, but did not exclude early sinusoidal injury.
Spleen assessment	Retrospective restaging CT review showed no interval splenomegaly. Craniocaudal splenic length was approximately 11 cm and unchanged. Volumetric segmentation was not performed.	The absence of new splenomegaly reduced diagnostic certainty for established oxaliplatin-induced SOS.
Inflammatory profile	CRP 6.3 mg/L on admission, 5.2 mg/L on day 3; procalcitonin 0.163 ng/mL.	Favored low-grade inflammation rather than sepsis.
Microbiology	Urine culture grew ESBL-producing Escherichia coli and Enterococcus faecalis.	Colonization or localized inflammation was possible, but infection was unlikely to be the primary bleeding mechanism.
Portal-phase CT	Biliary stent and periportal or pancreatic tumor-related changes (Fig. 1A); venous collaterals adjacent to the conduit (Fig. 1B).	Suggested portal-hypertensive collateralization near the bleeding site.
Urographic CT	Circumferential conduit-wall thickening (Fig. 1C); nondilated upper urinary tracts without radiologic recurrence (Fig. 1D).	Favored conduit-wall inflammation and argued against obstructive upper-tract or recurrent-tumor bleeding.
Looposcopy	Diffuse inflammatory and friable mucosal changes in the Bricker loop; no focal tumor, stone, or biopsy.	Supported conduit inflammation, but mucositis remained unconfirmed histologically.
Doppler ultrasonography	Portal vein diameter 17 mm with preserved hepatopetal flow; splenic venous enlargement; no imaging features of cirrhosis.	Supported noncirrhotic portal-hypertensive physiology.
Excluded alternatives	No stones, hydronephrosis, focal arterial blush, or convincing luminal lesion.	Reduced the likelihood of common urologic causes of conduit bleeding.

*Abbreviations*: *CRP* C-reactive protein, *CT* Computed tomography, *ESBL* Extended-spectrum beta-lactamase, *SOS* Sinusoidal obstruction syndrome

Contrast-enhanced computed tomography of the abdomen and pelvis performed on admission redirected the differential diagnosis. Portal-phase images showed a biliary stent and periportal or pancreatic tumor-related changes (Fig. 1A) together with venous collaterals adjacent to the conduit (Fig. 1B). Urographic reconstructions showed circumferential thickening of the Bricker loop wall (Fig. 1C) but nondilated upper urinary tracts and no radiologic evidence of recurrent urothelial carcinoma or another upper-tract source (Fig. 1D). No urolithiasis, hydronephrosis, focal arterial blush, or convincing intraluminal tumor was identified. Retrospective review of restaging computed tomography showed no interval splenomegaly compared with earlier imaging. The craniocaudal splenic length was approximately 11 cm and unchanged. Formal volumetric splenic segmentation was not performed.

Looposcopy, defined as endoscopic inspection of the Bricker ileal conduit, was performed after initial stabilization. It showed diffusely inflamed and friable conduit mucosa without a focal tumor, stone, or obvious traumatic stomal lesion. No histopathologic specimen was taken because the bleeding had slowed, the suspected lesion was diffuse rather than focal, and biopsy was judged unlikely to change immediate management. The looposcopic appearance therefore supported conduit mucosal inflammation, but it did not provide histologic proof of chemotherapy-associated mucositis.

Doppler ultrasonography later during the same admission demonstrated a dilated portal vein measuring 17 mm, splenic venous enlargement, preserved hepatopetal flow, and no sonographic features of cirrhosis. The liver appeared steatotic rather than cirrhotic. The combination of portal vein dilatation, peri-conduit collaterals, and preserved hepatopetal flow supported noncirrhotic portal-hypertensive physiology, but portal venous pressure, hepatic venous pressure gradient, and catheter venography were not measured during the index admission.

The differential diagnosis is summarized in Table 3. Recurrent urothelial carcinoma, secondary conduit tumor, infection, stones, and local stomal trauma were considered but were not supported by the imaging pattern, looposcopy, or clinical course. The most cautious explanation was mixed-mechanism hemorrhage. Portal hypertensive stomal varices were the hemodynamic substrate. The underlying portal physiology may have reflected pancreatic tumor-related venous distortion, early oxaliplatin-associated sinusoidal injury, or both. Presumed irinotecan-associated conduit mucosal injury likely acted as an immediate trigger because bleeding occurred 8 days after the most recent cycle and looposcopy demonstrated diffuse mucosal inflammation.

**Table 3:** Differential diagnosis of Bricker conduit bleeding

Candidate diagnosis	Case-specific arguments for	Case-specific arguments against	Overall likelihood in this case
Recurrent urothelial carcinoma	History of bladder cancer and prior radical cystectomy.	Upper urinary tracts were nondilated; CT showed no radiologic recurrence around the conduit or upper tracts; looposcopy did not show a focal tumor.	Low
Secondary conduit neoplasm	Any bowel conduit can develop a secondary tumor over time.	No focal enhancing mass or luminal lesion was identified; bleeding was abrupt after chemotherapy rather than progressive.	Low
Infection-related hemorrhage	Positive urine culture and conduit bacteriuria.	Low CRP and procalcitonin, no sepsis, no fever, and no imaging evidence of pyelonephritis or abscess.	Low to intermediate
Stone disease or obstruction	Gross hematuria can accompany calculi or obstructive mucosal injury.	No urolithiasis or hydronephrosis on CT; upper urinary tracts remained nondilated.	Low
Local stomal trauma or technical lesion	Bleeding from a stoma may follow appliance trauma or a mucocutaneous lesion.	Imaging showed deeper peri-conduit venous collaterals and conduit-wall thickening; looposcopy did not show an isolated traumatic lesion.	Low
Portal hypertensive stomal varices	Peri-conduit collaterals on portal-phase CT and portal-vein dilatation with preserved hepatopetal flow.	No direct catheter venography, portal pressure measurement, or active variceal extravasation was obtained during the index admission.	High as a substrate; active bleeding not proven
Oxaliplatin-associated sinusoidal injury or SOS	Oxaliplatin exposure, portal vein dilatation, noncirrhotic liver morphology, and ectopic collaterals.	Only two cycles; reconstructed cumulative oxaliplatin exposure approximately 170 mg/m²; no new splenomegaly; platelet counts remained normal; no liver histology.	Possible contributor, not proven
Irinotecan-associated conduit mucositis	Bleeding occurred 8 days after irinotecan; CT showed circumferential conduit-wall thickening; looposcopy showed diffusely inflamed and friable conduit mucosa.	No histologic confirmation was obtained.	Probable mucosal co-trigger
Mixed mechanism: varices plus mucositis	Best fit for chronology, CT, looposcopy, portal hemodynamics, and absence of another source.	Relative contribution of each component cannot be quantified retrospectively.	Most likely overall interpretation

*Abbreviations*: *CRP* C-reactive protein, *CT* Computed tomography, *SOS* Sinusoidal obstruction syndrome

The patient received tranexamic acid, etamsylate, intravenous corticosteroids followed by oral prednisone taper, propranolol, red blood cell transfusion, hydration, and antimicrobial therapy. These measures should not be interpreted as definitive treatment of variceal hemorrhage. They were used for stabilization and for the suspected inflammatory and infectious components while the bleeding stopped. Hematuria ceased and hemoglobin stabilized. He was discharged later in February 2026 with oncology and urology follow-up, planned reconsideration of further irinotecan exposure, and awareness that recurrent bleeding would mandate early interventional-radiology reassessment. At last follow-up, conduit bleeding had not recurred. The patient’s overall condition remained chronically poor, with poor ECOG performance status attributed to the underlying pancreatic cancer. The patient’s own perspective on this episode was not available from the contemporaneous record.

### Discussion and conclusions

This case is clinically important because it shows that severe Bricker-conduit bleeding should not be framed as an exclusively urologic event. The standard differential diagnosis remains correct and must be addressed first, because recurrent urothelial carcinoma, late conduit tumors, infection, calculi, and technical stomal lesions are all real causes of hemorrhage after urinary diversion [1–3]. However, when blood loss is abrupt, high-volume, or poorly explained by routine urinary tract findings, a conduit fashioned from ileum should also be regarded as ectopic bowel and therefore as a potential site of portal-hypertensive collateralization [4–7]. In our patient, portal-phase computed tomography, Doppler ultrasonography, and looposcopy were complementary. None was sufficient alone.

The portal-hypertensive substrate was plausible even without cirrhosis. Pancreatic malignancy can produce noncirrhotic, segmental, or mixed portal hypertension through venous compression, invasion, thrombosis, or periportal desmoplastic distortion [16, 17]. In this context, the combination of a dilated portal system, preserved hepatopetal flow, conduit-adjacent venous collaterals, and absence of another convincing urinary-tract source strongly favored ectopic stomal varices as a clinically relevant finding. This interpretation also explains why conventional local hemostasis may fail: the pressure gradient driving the collateral remains unchanged unless the venous pathway itself is interrupted or decompressed [4, 5, 7].

The role of oxaliplatin must be considered carefully. Oxaliplatin-induced sinusoidal obstruction syndrome is a well-recognized cause of noncirrhotic portal hypertension in oncologic patients [9, 10]. This possibility is therefore clinically important. In the present case, standard-dose reconstruction indicated only two FOLFIRINOX cycles and a cumulative oxaliplatin exposure of approximately 170 mg/m², or approximately 310 mg total. Platelet counts remained within the reference range, and retrospective computed tomography review did not show new splenomegaly. These features argue against confidently labeling the case as established, typical oxaliplatin-induced sinusoidal obstruction syndrome. They do not exclude early sinusoidal endothelial injury or portal sinusoidal vascular disease. For that reason, the revised interpretation treats oxaliplatin-associated sinusoidal injury as a plausible co-driver of portal hypertension rather than as a proven single cause.

Portal hypertension alone, however, may not fully explain the timing of this episode. The hemorrhage began 8 days after irinotecan exposure, within the expected window for delayed irinotecan toxicity [14, 15]. Guidance from the Multinational Association of Supportive Care in Cancer and the International Society of Oral Oncology recognizes gastrointestinal mucositis as a systemic toxicity of cancer therapy, but the literature offers almost no discussion of how such injury should be recognized when the exposed bowel segment has been repurposed as a urinary diversion [12, 13]. The absence of diarrhea in our patient does not argue strongly against irinotecan-related injury because the conduit is excluded from enteric continuity. In this setting, circumferential conduit-wall thickening (Fig. 1C), diffuse inflammatory changes on looposcopy, low-grade inflammatory markers, lack of sepsis, and tight chronology with chemotherapy are more informative than bowel symptoms alone.

The response to conservative therapy also needs careful interpretation. Tranexamic acid, etamsylate, corticosteroids, hydration, transfusion, antimicrobial therapy, and propranolol are not definitive acute treatments for ectopic variceal hemorrhage. Their apparent association with cessation of bleeding does not prove that varices were the active bleeding point. It is possible that ectopic varices were present as a portal-hypertensive substrate and that the dominant immediate lesion was inflamed, friable conduit mucosa. It is also possible that varices bled intermittently and stopped spontaneously after stabilization. Because portal pressure and catheter venography were not obtained, the relative contribution of each mechanism cannot be quantified. The term mixed mechanism is therefore intentionally cautious.

The published literature supports the need for early portal-venous assesment but also highlights how rarely this entity is recognized. The 2024 review by Xu and colleagues identified only 27 accessible cases of stomal variceal hemorrhage in ileal conduit diversion [6]. Subsequent and earlier reports describe hemostasis using transjugular transhepatic embolization, N-butyl cyanoacrylate embolization through a recanalized paraumbilical vein, transportal selective angioembolization, embolization of subcutaneous mesenteric varices, and direct percutaneous embolization with gelatin sponge and ethanolamine oleate [18–22]. These reports collectively show that once the culprit collateral is defined, catheter-based therapy can be effective, but technique must be individualized to venous anatomy and access route.

For that reason, conservative stabilization in the present case should be understood as temporizing rather than definitive. A practical escalation framework is summarized in Table 4. If bleeding recurs, the first step should be urgent portal-phase computed tomography or computed tomography angiography with venous mapping, followed by catheter venography and pressure assessment when technically appropriate. If a discrete peri-conduit collateral can be mapped, selective embolization or sclerotherapy is the most direct escalation [7, 18–22]. If imaging shows a treatable portal or mesenteric venous stenosis, portal venous stenting can be rational salvage therapy, particularly in cancer-related anatomy [23]. If the physiology is dominated by left-sided portal hypertension or splenic inflow, partial splenic embolization may be considered in selected patients [16, 24]. When anatomy and prognosis permit, portal decompression with transjugular intrahepatic portosystemic shunt or another shunt strategy remains the most durable option because it addresses the driving pressure rather than the bleeding endpoint [7].

**Table 4:** Escalation options if hemorrhage recurs

Strategy	Main target	When to consider	Main limitations or notes
Conservative therapy and observation	Temporary hemostasis and stabilization	Reasonable only when bleeding stops, there is no persistent shock, and immediate invasive risk outweighs benefit.	Not definitive for variceal hemorrhage; recurrence risk persists if portal inflow remains pressurized.
Urgent portal-phase CT or CT angiography with venous mapping	Definition of inflow, outflow, and culprit collateral anatomy	First-line reassessment if any recurrent high-volume bleeding occurs.	Should be planned with interventional radiology; standard urographic imaging alone may be insufficient.
Catheter venography with pressure assessment	Direct confirmation of venous anatomy and pressure gradient	When imaging suggests a treatable collateral, stenosis, or portal-systemic pathway.	Invasive; may be inappropriate in poor performance status or limited oncologic prognosis.
Selective embolization or sclerotherapy	Culprit peri-conduit varix or mesenteric collateral	Preferred next escalation if rebleeding occurs and a target vessel is defined.	May not be durable if the underlying portal-hypertensive circuit remains pressurized.
Portal decompression with TIPS or another shunt strategy	Driving portal pressure gradient	Most relevant when anatomy, liver function, and overall prognosis support durable decompression.	Suitability depends on portal anatomy, competing cancer prognosis, and procedural risk.
Portal venous stenting	Focal portal or mesenteric venous stenosis in cancer-related anatomy	Rational salvage when venous narrowing is demonstrable and technically accessible.	Best suited to selected patients with a clear venous target rather than diffuse collateralization.
Partial splenic embolization	Left-sided or splenic inflow-dominant portal hypertension	Useful only if portal-hemodynamic evaluation suggests a dominant splenic contribution.	Not relevant to every case; should be tailored to vascular anatomy.
Local conduit endoscopy or stomal intervention	Alternative focal intraluminal lesion	Useful if repeat evaluation suggests tumor, stone, ulcer, or a focal bleeding point inside the conduit.	Unlikely to solve venous bleeding driven by portal hypertension.
Conduit excision with bilateral cutaneous ureterostomies	Definitive removal of the bowel conduit from the urinary tract	Salvage option when bleeding remains conduit-centered and endovascular control is unlikely to last.	Requires major reconstructive decision-making and careful patient selection, particularly in poor ECOG status.

*Abbreviations*: *CT* Computed tomography, *ECOG* Eastern Cooperative Oncology Group, *TIPS* Transjugular intrahepatic portosystemic shunt

Urologists should also be prepared to discuss definitive reconstructive salvage. If the conduit itself becomes the recurrent bleeding locus and repeated endovascular control is unlikely to last, excision of the ileal conduit with conversion to bilateral cutaneous ureterostomies may be more rational than repeated admissions for transfusion and rescue hemostasis. Contemporary comparative studies, systematic reviews, and a recent meta-analysis indicate that cutaneous ureterostomy remains an accepted urinary diversion in frail or high-risk patients and may reduce operative burden relative to bowel-based diversion in selected settings [25–28]. In a patient with advanced pancreatic cancer, portal-hypertensive collateralization around an ileal segment, poor ECOG performance status, and anticipated future systemic therapy, undiversion should therefore be viewed as a legitimate salvage option rather than a purely last-ditch maneuver.

The main limitations are substantial and have been intentionally stated. First, looposcopy showed mucosal inflammation, but no biopsy was taken, so irinotecan-associated conduit mucositis remains presumed rather than proven. Second, portal pressure, hepatic venous pressure gradient, and catheter venography were not measured, so active variceal bleeding cannot be proven retrospectively. Third, oxaliplatin-associated sinusoidal injury is biologically plausible, but the short exposure, normal platelet trend, and absence of new splenomegaly prevent a firm diagnosis of established sinusoidal obstruction syndrome. Fourth, the short-term follow-up is reassuring because bleeding had not recurred by May 2026, but the patient’s poor oncologic condition limits the strength of long-term conclusions.

The take-home points are straightforward. Massive hematuria from a Bricker conduit may be multifactorial rather than monolesional. Portal-phase imaging should be obtained early when bleeding is disproportionate to routine urinary tract findings. Looposcopy can support mucosal inflammation, but biopsy is required for histologic confirmation when it can be performed safely. The coexistence of peri-conduit venous collaterals, conduit-wall inflammation, absence of upper-tract recurrence or obstruction, and high-volume stomal hemorrhage should prompt clinicians to consider a mechanism beyond tumor recurrence or infection alone. Early multidisciplinary planning can shorten the path from repeated transfusions to targeted embolization, portal decompression, venous stenting, or urinary undiversion.

## Data Availability

All data generated or analyzed during this study are included in this published article and its supplementary information files. Further de-identified details are available from the corresponding author on reasonable request, subject to patient privacy restrictions.

## Abbreviations

BSA: Body-surface area
CT: Computed tomography
ECOG: Eastern Cooperative Oncology Group
ERCP: Endoscopic retrograde cholangiopancreatography
ESBL: Extended-spectrum beta-lactamase
FOLFIRINOX: Fluorouracil, leucovorin, irinotecan, and oxaliplatin
HVPG: Hepatic venous pressure gradient
ISOO: International Society of Oral Oncology
MASCC: Multinational Association of Supportive Care in Cancer
SOS: Sinusoidal obstruction syndrome
TIPS: Transjugular intrahepatic portosystemic shunt
